# Tricuspid Valve Damage Related to Transvenous Lead Extraction

**DOI:** 10.3390/ijerph191912279

**Published:** 2022-09-27

**Authors:** Anna Polewczyk, Wojciech Jacheć, Dorota Nowosielecka, Andrzej Tomaszewski, Wojciech Brzozowski, Dorota Szczęśniak-Stańczyk, Krzysztof Duda, Andrzej Kutarski

**Affiliations:** 1 Institute of Medical Sciences, Jan Kochanowski University, 25-369 Kielce, Poland; 2 Department of Cardiac Surgery, Świętokrzyskie Center of Cardiology, 25-736 Kielce, Poland; 3 2nd Department of Cardiology, Faculty of Medical Sciences in Zabrze, Medical University of Silesia in Katowice, 41-800 Zabrze, Poland; 4 Department of Cardiology, The Pope John Paul II Province Hospital, 22-400 Zamość, Poland; 5 Department of Cardiology, Medical University of Lublin Poland, 20-059 Lublin, Poland; 6 Department of Cardiac Surgery, Masovian Specialistic Hospital, 26-617 Radom, Poland

**Keywords:** transvenous lead extraction complications, tricuspid valve damage, risk factors, long-term survival

## Abstract

Background: Damage to the tricuspid valve (TVD) is now considered either a major or minor complication of the transvenous lead extraction procedure (TLE). As yet, the risk factors and long-term survival after TLE in patients with TVD have not been analyzed in detail. Methods: This post hoc analysis used clinical data of 2631 patients (mean age 66.86 years, 39.64% females) who underwent TLE procedures performed in three high-volume centers. The risk factors and long-term survival of patients with worsening tricuspid valve (TV) function after TLE were analyzed. Results: In most procedures (90.31%), TLE had no negative influence on TV function, but in 9.69% of patients, a worsening of tricuspid regurgitation (TR) to varying degrees was noted, including significant dysfunction in 2.54% of patients. Risk factors of TLE relating to severe TVD were: TLE of pacing leads (5.264; *p* = 0.029), dwell time of the oldest extracted lead (OR = 1.076; *p* = 0.032), strong connective scar tissue connecting a lead with tricuspid apparatus (OR = 5.720; *p* < 0.001), and strong connective scar tissue connecting a lead with the right ventricle wall (OR = 8.312; *p* < 0.001). Long-term survival (1650 ± 1201 [1–5519] days) of patients with severe TR was comparable to patients without tricuspid damage related to TLE. Conclusions: Severe tricuspid valve damage related to TLE is relatively rare (2.5%). The main risk factors for the worsening of TV function are associated with a longer lead dwell time (more often the pacing lead), causing stronger connective tissue scars connecting the lead to the tricuspid apparatus and right ventricle. TVD is unlikely to affect long-term survival after TLE.

## 1. Introduction

Transvenous lead extraction (TLE) is currently the main part of the lead management strategy [1,2,3,4,5]. Connective scar tissue surrounding the lead and binding it to heart structures creates a risk of venous or cardiac wall injury with severe bleeding [6,7,8,9,10], or severe damage to the tricuspid valve (TVD) [11,12,13,14,15,16,17,18,19,20]. The TVD was omitted in previous guidelines [1,2,3,4] as a significant complication of TLE and was only just considered more recently [4,5]. Attempts to search for the risk factors of major complications were started many years ago [21,22,23,24,25,26,27,28,29,30], but lead extraction-related tricuspid valve damage was omitted or considered marginal. The clinical implications of TVD relating to TLE are not fully understood thus far, and the problem has not largely been analyzed in research material. Most reports so far have been based upon small study populations (40–206 patients) [11,12,13,14,15,16,17,18,19,20] and their results are inconclusive. According to some studies, exacerbation of tricuspid regurgitation associated with TLE may lead to longer hospital stays, right-sided heart failure and may worsen long-term prognosis [11,13,19], while other reports show mostly mild, or insignificant damage [14,15,17,18]. The aim of the present study, based on a large database of patients undergoing TLE, was to evaluate the incidence, risk factors, and the impact of TVD on the clinical status and long-term prognosis of patients. Additionally, the current work presents the possibilities of preventing significant damage to the tricuspid valve during TLE.

## 2. Methods

### 2.1. Study Population

This post hoc analysis used clinical data from 2631 patients who underwent transvenous lead extraction between November 2007 and September 2021 in three high-volume centers. All procedures were performed by the same first operator in cooperation with three experienced cardiac surgeons, three anesthesia teams and four experienced echocardiographers. All information concerning patients and procedures were up-to-date and inserted to computer database prospectively.

### 2.2. Lead Extraction Procedure-Definitions

Lead extraction procedures were defined according to the most recent guidelines on the management of lead-related complications (HRS 2017 and EHRA 2018) [4,5]. Indications for TLE and the type of periprocedural complications were defined according to the 2017 HRS Expert Consensus Statement on Cardiovascular Implantable Electronic Device Lead Management and Extraction [4]. Definitions for lead extraction complete procedural success, clinical success, failure of lead extraction, non-functional lead, abandoned lead, major and minor complications, and remnant of “small” residual lead portion were determined according to the 2017 Heart Rhythm Society Expert Consensus paper on transvenous lead extraction [4].

### 2.3. Indications for Lead Extraction

TLE indications were divided into: (1) infectious, consisting of a localized pocket infection, bacteremia with or without endocarditis, or any combination of these presentations together; and (2), non-infectious: mechanical lead damage (electric failure), lead dysfunction (exit/entry block, dislodgement, extracardiac pacing, perforation, upgrading, downgrading), and other reasons for preventing lead abandonment (atrial fibrillation, pooling of leads), threatening/potentially threatening lead (free ending, left heart, lead-dependent TVD), other (MRI indication, cancer, pain of pocket, loss of indication for pacing/ICD) and recapture of venous access (symptomatic occlusion, sinus vena cava syndrome, lead replacement/upgrading).

Indicators of increased procedure complexity included: unexpected procedure difficulties, so called “technical problems”, block-in-lead at the venous entry (subclavian region), lead-to-lead strong connection with scar tissue leading to the difficult separation of the two leads, Byrd dilator collapse/torsion, lead break during extraction, necessity to change venous approach, loss of free lead fragment, and the necessity to utilize second line tools (metal sheath, Evolution TightRail, lasso catheter/snare or basket catheter, or Femoral Work Station) [30].

### 2.4. Lead Extraction-Techniques

Whenever possible, the primary approach was the implant vein access. In cases where the proximal lead ended inside the cardiovascular system (CVS), or where a lead was broken during the extraction, the femoral, jugular, or subclavian recaptured lead Venous Entry Approach was utilized [8,10,20,30]. Standard stylets were used to stiffen the leads in most cases, excluding situations where the ending of the lead was not available. However, if the lead was long enough to be retrieved via any entry, the stylet was inserted to continue the procedure. The locking stylets (Locking, Cook^®^, Bloomington, IN, USA) were used only for extraction of the oldest leads with a high estimated risk of breakage. Laser and electrosurgical energy-powered sheaths were not used.

A superior approach (lead venous entry), using non-powered mechanical systems with various stylets and polypropylene telescoping (Byrd) dilatators (Cook^®^), was always the first-line technique for lead extraction. A powered mechanical sheath system (Evolution, Cook; TightRail Spectranetics) was only used if the polypropylene telescoping sheaths appeared ineffective. A femoral approach, using the Femoral Work Station with a basket, the Amplatz GooseNeck^®^ Snare Kit (Amplatz, Roseville, MI, USA), and occasionally, Byrd dilators, were used for free-floating leads with proximal endings in the lumen of the CVS [8,10,20,30].

The organizational model of TLE procedures changed from lead extraction in EPS-LAB with intravenous deep sedation/analgesia (2007–2011) via staging of safety precautions (most difficult procedures in the operating room under general anesthesia) (2012–2015) up to all procedures performed in a hybrid operating room, under general anesthesia, TEE monitoring and cardiac surgeon presence as co-operator (since 2015) [8,10,20,30].

### 2.5. Echocardiographic Examinations

Routine transthoracic echocardiography (TTE) was mandatory as a pre- and post-procedural examination, as well as a transesophageal (TEE) pre- and post-procedural examination. Patients with incomplete echocardiographic examinations were excluded from the analysis. In 1222 patients, the TLE procedure was additionally monitored using TEE [31,32,33,34]. In the remaining 1409 patients, preoperative and postoperative examinations were performed in the hybrid room just before and after the TLE procedure. The mid-esophageal, inferior esophageal and modified transgastric views were utilized to visualize the right heart chambers and the tricuspid valve [34]. In order to obtain a complete visualization of the anatomical structures and to fully assess the course of the leads, non-standard imaging planes were sometimes required [31,32,33,34].

Continuous transesophageal echocardiography monitoring or pre- and postoperative TEE in our series was performed using Philips iE33 or GE Vivid S 70 machines equipped with X7-2t Live 3D or 6VT-D probes. All recordings were archived, which enabled exact comparison (pre- and postoperative) of tricuspid valve (TV) condition and chordae tendineae [31,32,33,34]. Thanks to continuous monitoring, it was possible to elucidate the mechanism for the observed fall in blood pressure during TLE [31,32,33,34]. The retraction of the right ventricular (RV) wall was visible in 2D, TEE was confirmed in 3D imaging, and the cause of the pressure fall could be quickly identified. Furthermore, it is also important for the operator to control the simultaneous pulling on the other lead in case of lead-to-lead binding.

### 2.6. Tricuspid Valve Damage Evaluation

To describe changes in tricuspid valve (TV) regurgitation before and after TLE, we used standard parameters for the evaluation of TV severity, as recommended by the European Association of Echocardiography [35]. Additional analysis concerning all damages of subvalvular apparatus and the break of chordae tendineae were noted separately as additional phenomenon.

According to ESC recommendations, we defined four degrees of tricuspid regurgitation (TR): none, small, moderate, and severe. TR small/trivial/mild-color flow jet: 1 i 2; CW jet: faint/parabolic; VC ≤ 3 mm. TR moderate-color flow jet: 3; CW jet: dense/parabolic; VC > 3 and <7 mm. TR severe-color flow jet: 4; CW jet: dense/triangular with early peaking (peak < 2 m/s in massive TR); VC ≥ 7 mm.

To evaluate the influence of TLE on TV function, we assumed a slight (non-significant) impairment of TV function with only an increase of TR in one degree, and a significant worsening of TV function with an increase in TR of 2 or 3 degrees.

### 2.7. Statistical Analysis

According to the study protocol, the patients were divided into three groups, depending on the change in the degree of tricuspid regurgitation after TLE; group A with an increase by at least 2 degrees, group B with an increase by 1 degree, group C with no worsening of TV function after TLE (control group). The distribution of continuous variables in each group were estimated using the Shapiro–Wilk test. Some of the examined variables presented non-normal distribution. For uniformity, all continuous variables were presented as the mean ± standard deviation. The categorical variables were presented as a number and a percentage. Due to the large disproportion in the size of the groups (A, B vs. C), non-parametric tests were used for comparison: Chi2 test with Yates correction (dichotomous data), or unpaired “U” Mann–Whitney test (continuous data). The comparison was performed between groups A vs. C, B vs. C and A vs. B. To determine which parameters influenced the change in the degree of TVR during TLE, multivariable stepwise linear regression analysis was used. A significance level of *p* ≤ 0.05 (U Mann–Whitney or Chi2 analysis) was required to include a variable in the model, and a significance level of *p* ≤ 0.1 was required for a variable to remain in the model (from highly correlated data, only one was included in the model).

The analyses were performed for an increase in TVR by 1 degree and for an increase by at least 2 (or 3) degrees. 

A *p*-value less than 0.05 was considered statistically significant. Statistical analysis was performed with Statistica version 13.3 (TIBCO Software Inc., Palo Alto, CA, USA).

### 2.8. Approval of the Bioethics Committee

All patients gave their informed written consent to undergo TLE and for the use of anonymous data from their medical records, as approved by the Bioethics Committee at the Regional Chamber of Physicians in Lublin No. 288/2018/KB/VII, and the consent process was performed according to the principles expressed in the Declaration of Helsinki.

## 3. Results

### 3.1. Clinical Data of Study Group

The study population contained 2631 patients, with a mean age of 66.86 years, including 39.64% females. The clinical parameters were as follows: average left ventricular ejection fraction (LVEF) was observed in 49.49% of patients, renal failure was observed in 21.10% of patients, ischemic heart disease was observed in 58.68% of patients, and mean Charlson’s comorbidity index was 4.82 ± 3.69 points. Indications for TLE included: systemic infection (with pocket infection or not) in 23.68% of patients, local (pocket) infection in 7.72% of patients, lead failure (replacement) in 50.45% of patients, change of pacing mode/upgrading, downgrading in 8.16% of patients, and other indications in 18.07% of patients. The types of implanted devices were as follows: pacemaker (all types) in 70.62% of patients, ICD in 21.74% of patients, and CRT-D in 7.64% of patients. Average dwell time of oldest lead in the patient before TLE was 104.8 months, and the average cumulative dwell time of leads before TLE was 15.85 years.

### 3.2. Analysis of Data on the Function of the Tricuspid Valve

Analysis of the changes to the degree of TR after transvenous lead extraction showed that in most patients (2376, 90.23%), there was no impairment of TV function after TLE, but in 255 patients (9.69%), a different degree of tricuspid regurgitation increase was observed. Significant worsening (by at least 2 degrees) was not so frequent (67 pts, 2.55%). The predominant form of TLE-related TVD was an increase in TR by 1 degree (188, 6.41%). Most patients with significant TV damage had a degree meeting criteria for surgical correction. In two (0.08%) patients, the rescue/immediate plastic repairs of the TV by leaflet sutures were carried out. In another ten patients (0.38%), delayed TV plastic repair was performed as a planned operation. In the next eight patients (0.30%), due to improvement in TV function within several months after TLE, the operation was temporarily postponed and patients remained under observation. Several patients refused operation (Table 1).

The analysis of the risk factors of TVD related to TLE showed that a significant worsening of TV function during TLE was usually connected to a younger patient’s age during TLE and during the first system implantation, higher LVEF, a rare appearance of heart failure (NYHA III & IV class), a low incidence of permanent atrial fibrillation, a lower Charlson’s index and a higher risk of infectious complications according to the PADIT score [36]. Among the factors relating to the implanted system, it was shown that a significant deterioration in valve function was found more frequently in patients with a pacemaker device (type AAI, VVI, DDD, CRT-P), the presence and number of abandoned leads before TLE, and a longer dwell time of the oldest lead in the patient. A lower incidence of significant TV damage during TLE was observed in patients with an ICD device (VVI, DDD). Differences of analyzed parameters within the biggest group without changes in TR after TLE, in a subgroup with non-significant worsening of TR similarly to factors analyzed in Table 2, were less visible, but the tendency was similar (Table 2). 

The analysis of the influence of the complexity of TLE on tricuspid valve function showed that a longer procedure duration, a higher number of extracted leads, a strong lead-to-lead connection with connective scar tissue, the appearance of any technical problems during lead extraction, the necessity for the use of second-line tools (Evolution or TightRail, or lasso catheter/snare catheter), the extraction of pacemaker leads (especially unipolar leads, leads with a passive fixation, or extraction of an abandoned lead), and the longer dwell time of extracted leads, were all factors which were demonstrably connected to the significant worsening of TV function. The risk of major complications calculated by SEFETY TLE calculator [25], www.usuwanieelektrod.pl (accessed on 27 August 2022) before lead extraction was definitely higher in the group with further TV dysfunction.

**Figure 1:**
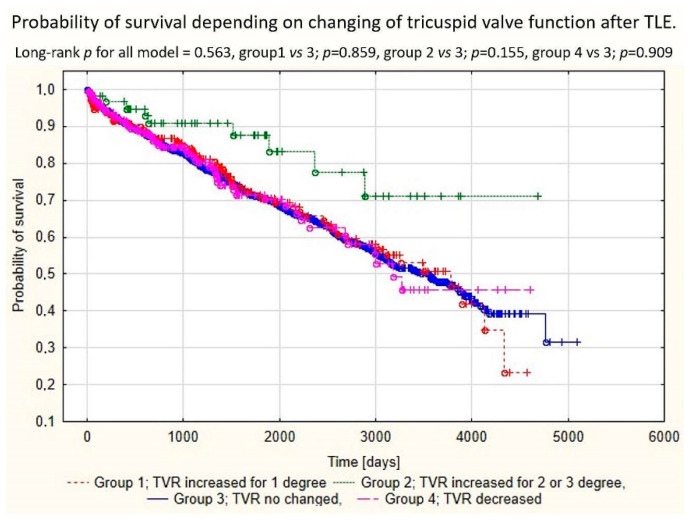
Kaplan–Meier survival curves depending on the presence of TLE-related TVD.

A comparative analysis of the efficacy of TLE in the analyzed groups of patients showed a connection between the appearance of significant TLE-related TVD and incomplete lead extraction (appearance of partial radiological success), and the occurrence of other major complications. The percentage of full clinical success and full procedural success were markedly lower in patients in which significant TV damage was noted. One of other reasons for the phenomenon was that if serious TV damage reached criteria for cardiac surgery, it was considered a major complication. The appearance and severity of TLE-related TVD seems to have no clear influence on long-term survival after lead extraction. Long-term mortality after TLE is probably related to factors other than TV damage. During 1650 ± 1201 [1–5519] days follow-up, a lower mortality in patients with increased TR was detected (Table 3, Figure 1).

The analysis of preoperative echocardiographic findings showed a lot of factors which were clearly connected to the increased risk of significant TV damage during TLE. The most important were: a higher LVEF, a lack of significant or severe TR before TLE, the presence of any shadows on the leads before TLE, thickening of the lead, presence of any strong connective scar tissue connections between the lead and heart structures or with another lead, and the presence of abnormally long lead loops in the heart. Monitoring of the lead extraction procedure with TEE allowed for the recognition of an alarming phenomenon which frequently precedes TV damage, namely the drawing on a tricuspid leaflet or RV wall during mechanical lead extraction. Similar findings, but less clearly escalated, were noted in patients with non-significant TV damage during lead extraction. In conclusion, significant TV damage appears most frequently in a normal heart, without significant or severe TR, and in the presence of intensive scar growth, where there is the binding of a lead with heart structures or leads inter se (Table 4).

Multivariable regression showed that the most important prognostic factors of mild TVD during TLE were: the presence of an abandoned lead (OR = 1.624; *p* = 0.048), strong connective scar tissue connecting the lead with tricuspid apparatus (OR = 3.452; *p* < 0.001) and lead loops in the heart (OR = 1.726; *p* = 0.003). Tricuspid valve regurgitation before TLE reduces the likelihood of further aggravation of regurgitation by 45.40% (*p* < 0.001) per each degree.

The prognostic factors of severe TVD during TLE were: the extraction of leads of conventional pacemakers (OR = 5.246; *p* = 0.029), dwell time of the oldest extracted lead (OR = 1.076; *p* = 0.032), strong connective scar tissue connecting lead(s) with tricuspid apparatus (OR = 5.720; *p* < 0.001) and strong connective scar tissue connecting lead(s) with the right ventricle wall (OR = 8.312; *p* < 0.001). The presence of TR before TLE was connected with a lower probability of worsening TV function during TLE (OR = 0.155 per each degree; *p* < 0.001) (Table 5).

## 4. Discussion

Transvenous lead extraction is an integral part of the management of pacing or ICD lead-related problems [1,2,3,4,5]. TLE is currently considered as a relatively safe procedure: the risk of major complications according to the available literature is 0.4–3.4%; the risk of death, 0.00–1.86% [1,2,3,4,5,7,9]. Damage to the tricuspid valve during lead extraction is estimated to be in the range of 3.5% to 15%, and even up to 19% [4,5,12,13,14,15,16,17,18,19]. Literature and guidelines on this subject concentrate on cardiovascular wall tear, but not on TLE-related worsening of TR [1,2,3,4,5,7,9]. In 2018, an EHRA expert consensus statement on lead extraction [5] found much lower percentages of tricuspid valve damage related to TLE: flail tricuspid valve leaflet requiring intervention, 0.03% (being a major complication), and worsening tricuspid valve function, 0.02–0.59% (being a minor complication). The present study showed that in 90.31% of patients, TLE had no negative influence on TV efficacy, in 9.70% of patients, a different worsening degree of tricuspid regurgitation was noted, but significant worsening (by two or three degrees) was rare (2.54%). It is probable that such large discrepancies in the assessment of the incidence of TVD during TLE results from the frequent lack of echocardiographic monitoring of the procedure. Therefore, the present study is likely to provide a more realistic picture of the incidence of tricuspid valve damage during TLE.

**Figure 2:**
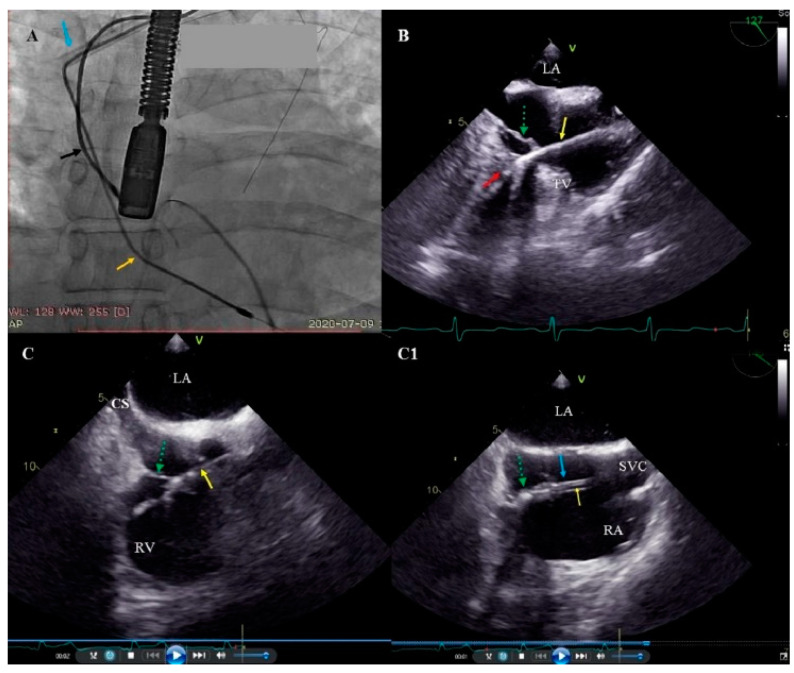
Pulling-up of the tricuspid valve (TV) leaflet while removing the lead adhered to the tricuspid apparatus. (**A**) Fluoroscopy. Initial phase of the removal of the ventricular lead (yellow arrow) using the Byrd dilator (blue arrow). Visibly tense ventricular lead. Atrial lead (black arrow). (**B**) 2D transesophageal echocardiography (TEE), mid-esophageal view. Simultaneous TEE image. Pulled-up ventricular lead (yellow arrow) with uncontrolled pulling of the septal leaflet of the TV (green arrow) lead adhesions to the septum (red arrow). (**C**,**C1**) 2D TEE, middle and low esophageal views. The next steps in removing the adherent to the posterior TV leaflet (green arrow) of the ventricular lead (yellow arrow). (**C1**) Winding of the leaflet (green arrow) on the dilator (blue arrow).

There were a few reports concerning TLE-related TVD, and little is known about the risk factors for this complication [11,12,13,14,15,16,17,18,19,20], only the role of the long duration of the implant and the pacemaker lead (as opposed to the ICD lead) is emphasized. Givon et al. reported the role of mechanical utility tools and a younger age [15], Coffey et al.—the role of age ≥75, extraction of ≥2 leads, powered sheath-assisted extraction, and pacemaker leads [17], Park et al.—advanced lead age and pacemaker [13], Roeffel et al.—difficult extraction and when tools such as a laser sheath are necessary [12]. The present study showed that the most important predictive factors of TLE-related TVD were: extraction of the leads of conventional pacemakers, dwell time of the oldest extracted lead and strong connective scar tissue binding the lead to the TV or the wall of right ventricle. It should be emphasized that the existing literature has not precisely assessed the impact of the presence of connective tissue adhesions with individual heart structures on the possibility of TLE-related TVD. Meanwhile, the detailed assessment of the location of lead growth and adhesion to the walls of the heart and veins presented in this study certainly allows the risks of valve damage to be predicted. The main mechanism of TV damage during mechanical lead dilatation is the attachment of the lead to the leaflet with scar tissue, but similarly important is excessive pulling of the extracted lead during the dilatation from venous or atrial levels, just before the dilatating sheath reaches the TV. It is our feeling and impression that mechanical lead dilatation from the venous route takes more effort and sometimes results in greater extracted lead pulling. This also appears to confirm the possibility that simultaneous lead traction from above and below during dilatation can also protect the TV [37]. This interesting idea needs further investigation (Figure 2).

**Figure 3:**
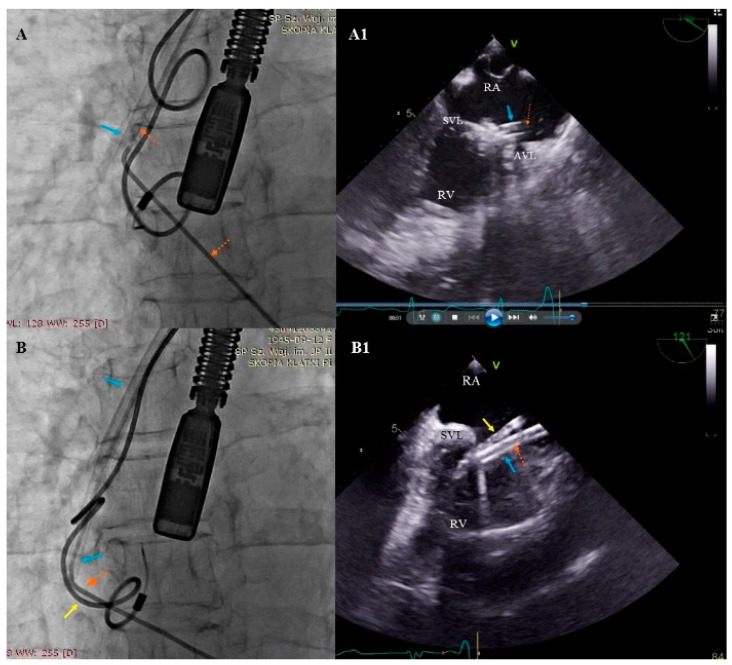
Winding of the leaflet on the dilatator while removing the ventricular lead (simultaneous images from fluoroscopy and transesophageal echocardiography (TEE). (**A**,**A1**) Tight ventricular lead (red arrow) and Byrd’s dilatator (blue arrow) winding the valve leaflets. (**B**,**B1**) When rotating the dilator in one direction (blue arrow), winding of the tricuspid valve (TV) leaflet and the attached atrial lead (yellow arrow) occurred (as a result of adhesions of the lead with the leaflet and inter-lead adhesions).

The second mechanism causing severe TV during TLE is the attachment of the leaflet to the lead with scar tissue and the predominant single direction sheath rotation which can create a winding of the TV leaflet on the dilatating sheath. This dangerous phenomenon cannot be seen during fluoroscopy, but is well visible by means of TEE monitoring. Warning information from the echocardiographer may prevent future disaster (Figure 3)

**Figure 4:**
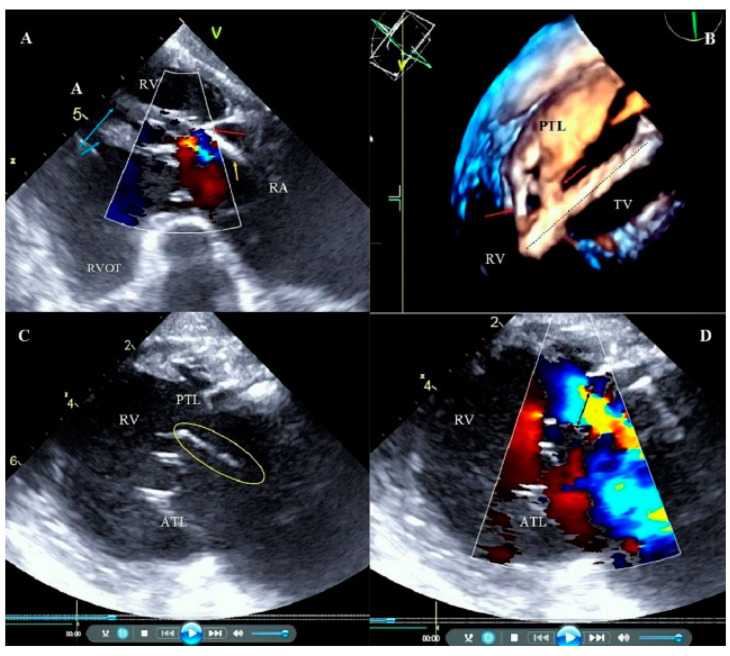
Adhesion of the lead to the tricuspid apparatus. Broken chordae tendineae during transvenous lead extraction (TLE). (**A**) 2D transesophageal echocardiography (TEE) transgastric view, color Doppler. The ventricular lead (yellow arrow) is fused with the posterior leaflet (red arrow) and the subvalvular apparatus. Papillary muscles (blue arrows). Low tricuspid regurgitation. (**B**) 3D TEE. Tricuspid valve (TV) view from the right ventricle (RV) side. Tissue bridges (adhesions) (red arrows) connecting the ventricular lead (dashed line) with the posterior leaflet (PTL). (**C**) 2D TEE transgastric view. Broken chordae tendineae (circle) that moves to right atrium (RA) (**D**) 2D TEE transgastric view. Image from panel C in the option of color Doppler, large TR to RA, Vena contracta (VC) = 11 mm (black arrow).

The analysis in the present study and analysis of previous reports [12,13,14,15,16,17,18,19,34] indicate that one of most important TLE safety challenges still remaining unsolved is the problem of TLE-related TVD which is caused by the adhesion of leads to TV leaflets with connective scar tissue. Significant lead pooling may disrupt the leaflet, but it appears that it is the mechanism of winding the leaflet on the dilatating sheath through rotation which causes it. Excellent co-operation with TEE monitoring may help warn the operator about potentially harmful situations leading to TV damage [31,32,33,34], but the extracted lead can be connected with chordae tendineae or even the head of the papillary muscle, and they can be damaged unnoticeably. The last question is an attempt to recommend a long-term lead management strategy. With respect to the prevention of TLE-related TV damage, the abandonment of unnecessary leads and excess leads in the patient should be avoided. This follows once again, the idea of the preventive replacement of leads after many years (when replacing the unit), even before their dysfunction or infection occurs. However, for many reasons, including organizational, it is too early to make such categorical statements (Figure 4).

The detailed analysis of the mechanisms of valve damage during TLE presented in the current study is certainly of significant importance in the prevention of this complication.

A very important issue is the further management of patients with valves damaged during TLE, and the impact of severe TR after TLE on long-term prognosis. In the present study we noted the worsening of TR for 1 degree in 7.15% of patients, for 2 degrees in 2.56% of patients, for 3 degrees in 0.58% of patients and severe dysfunction fulfilling criteria for cardiac surgery was found in 22 patients. In the group of patients with severe damage to the tricuspid valve, two patients (0.08%) required a rescue repair of the TV with leaflet sutures, and in another ten patients (0.380%), a TV repair was performed as a planned operation. The final eight patients (0.30%) remained under observation due to the improvement of the TV function. The need for surgical intervention was rare and comparable to previous reports [11,12,13,14,15,16,17,18,19,20,38]. It should be emphasized that the current study presents a very important aspect of the need for a thorough clinical and echocardiographic evaluation of patients showing a significant deterioration of valve function after TLE.

The present study also showed that the percentage of full clinical success and full procedural success were markedly lower in patients with significant TV damage, but no correlation was found between the worsening of TV function and long-term survival. The lack of influence of TLE-related TVD on long-term prognosis, as demonstrated in the present work, is certainly very important, especially with respect to doubts regarding lead management.

## 5. Conclusions

The incidence of significant damage to the tricuspid valve during transvenous lead extraction is approximately 2.5%, while the need for surgical intervention occurs only in 0.5% of these patients. The main risk factors of the worsening of TV function are associated with longer lead dwell time (more often the pacing lead) causing greater connective scar tissue connecting the lead to the tricuspid apparatus and the right ventricle. It is very important that the mechanism of valve damage is assessed by an experienced echocardiographer because it often prevents the development of severe regurgitation. Significant TLE-related TV damage has no influence on long-term survival after lead extraction.

### Study Limitations

There are some limitations of this study. All procedures were performed by a very experienced team and it may be difficult to repeat the obtained results in a small-volume center with a less experienced operator and team. All procedures were performed using all types of mechanical sheaths, but not laser powered sheaths. We examined only the effects of mechanical dilatation and we have no information regarding the use of laser energy for lead extraction and its influence on TV damage.

This study is a retrospective analysis of prospectively inserted database information. All information was collected from 2006 to 2021. In the period 2006–2014, TTE and TEE were performed before and after a TLE procedure, but in the period 2015–2021, additional TEE monitoring was routine. Due to technical reasons during TEE examinations, the evaluation of the quantitative parameters from the effective regurgitant orifice area (EROA) and the regurgitant volume (R vol), was not carried out.

## Figures and Tables

**Table 1:** Analysis of changes in the degree of tricuspid regurgitation after transvenous lead extraction.

	TR Before TLE	TR After TLE	Number of Patients	Percentage of 2631 Patoents	Worsening of TR in Degrees
All patients without worsening of TV function	0–4	0–4	2376	90.31%	0
Non-significant (for 1 degree) Impairment of TV function (7.16%)	0	1	27	1.03%	1
	1	2	87	3.31%	1
	2	3	52	1.98%	1
	3	4	22	0.84%	1
Significant (for 2 or 3 degrees) worsening of TV function 67 (2.55%)	0	2	3	0.11%	2
	1	3	43	1.63%	2
	2	4	9	0.34%	2
	0	3	1	0.04%	3
	1	4	11	0.42%	3
All patients with worsening of TV function			255	9.69%	
All examined patients			2631	100.0%	
Severe damage of TV during TLE reaching indications for cardiac surgery					
Tricuspid valve plastic repair performed immediately after TLE us rescue procedure			2	0.08%	
Tricuspid valve plastic repair performed as planned procedure			8	0.30%	
Tricuspid valve replacement			2	0.08%	
Borderline indications-observation only			8	0.30%	
Refused TV plastic repair-conservative treatment			3	0.11%	
Disqualification from TV plastic repair (cancer)			1	0.04%	
Other patients			2607	99.09%	

Abbreviations: TLE-transvenous lead extraction, TR-tricuspid regurgitation, TV-tricuspid valve.

**Table 2:** The comparison of patient-related, indication-related, system-related and history of pacing-related potential factors of TLE-related TVD.

Patient-Related Potential Risk Factors of TVDrTLE	TR Increased for 2 or 3 Degrees		TR Increased for 1 Degree		TR Unchanged or Decreased		“U” Mann-Whitney Ch^2^ Tests		
Number of Patients/Number of the Group	67	A	188	B	2376	C	*p* Values	*p* Values	*p* Values
Presented values	N/mean	Sd/%	N/mean	Sd/%	N/mean	Sd/%	A vs. B	A vs. C	B vs. C
Clinical characteristics									
Patient’s age during TLE [y]	62.42	19.28	66.58	14.46	67.01	14.83	0.038	0.397	0.756
Patient’s age during first system implantation [y]	47.34	21.77	55.97	16.17	58.67	15.82	0.029	<0.001	0.034
Sex (female)	31	46.27%	77	40.96%	935	39.32%	0.541	0.310	0.722
NYHA class III & IV	3	4.48%	20	10.64%	381	16.04%	0.207	0.017	0.063
LVEF < 40%	10	14.93%	43	22.72%	754	31.75%	0.230	0.005	0.012
Permanent AF	7	10.44%	49	26.06%	549	23.11%	0.013	0.022	0.382
Charlson’s index [points]	3.43	3.55	4.59	3.51	4.87	3.69	0.005	0.001	0.775
All infections indications	16	23.88%	68	36.17%	742	31.23%	0.092	0.251	0.186
PADIT score	5.62	2.72	5.24	2.86	4.76	2.81	0.316	0.021	0.010
All non-infective indications	51	76.12%	120	63.83%	1634	68.77%	0.092	0.251	0.186
System and history of pacing									
Device type-PM (AAI, VVI, DDD, CRT-P)	61	91.05%	149	79.26%	1648	69.36%	0.047	<0.001	0.006
Device type-ICD (VVI, DDD)	4	5.97%	32	17.02%	536	22.54%	0.043	0.001	0.095
Device type-CRT-D	2	2.99%	7	3.72%	192	8.08%	0.917	0.196	0.045
Presence of abandoned lead before TLE	15	22.39%	30	15.96%	234	9.85%	0.318	0.001	0.012
Number of abandoned leads before TLE	0.28	0.60	0.22	0.56	0.13	0.42	0.437	0.071	0.134
4 and >4 leads before TLE	3	4.48%	11	5.85%	64	2.69%	0.913	0.615	0.025
Dwell time of oldest lead in the patient before TLE [months]	181.3	87.23	127.7	72.41	100.9	74.64	<0.001	<0.001	<0.001

Abbreviations: AF—atrial fibrillation, AAI single chamber atrial pacing pacemaker. CRTP—cardiac resynchronization therapy pacemaker, DDD—dual chamber pacing pacemaker, ICD—implantable cardioverter defibrillator, LVEF: left ventricle ejection fraction, NYHA—New York Heart Association functional class, PM—pacemaker, TLErTVD—trans venous lead extraction tricuspid valve defect, TR—tricuspid regurgitation, [y]—[years], TR—tricuspid regurgitation, TLE—transvenous lead extraction, VVI—single chamber ventricle pacing pacemaker.

**Table 3:** Analysis of procedure complexity, efficacy, complications, outcomes and long-term mortality after TLE.

TLE Procedure Complexity, Efficacy, Complications, Outcomes and Long-Term Mortality After TLE	TR Increased for 2 or 3 Degrees		TR Increased for 1 Degree		TR Unchanged or Decreased		“U” Mann-Whitney Ch^2^ Tests		
Number of patients number of the group	67	A	188	B	2376	C	*p*	*p*	*p*
Presented values	N/mean	Sd/%	N/mean	Sd/%	N/mean	Sd/%	A vs. B	A vs. C	B vs. C
TLE procedure complexity									
Procedure duration (sheath to sheath) [minutes]	31.00	36.14	22.19	33.79	13.72	20.46	<0.001	<0.001	<0.001
Average time of single lead extraction (sheath-to sheath/number of extracted leads) [minutes]	18.39	24.14	11.59	14.77	8.02	11.37	0.005	<0.001	<0.001
Technical problem during TLE (any); n (%)	32	47.76%	55	29.26%	462	19.44%	0.010	<0.001	0.002
Two or more technical problems; n (%)	11	16.42%	10	5.32%	103	4.34%	0.005	<0.001	0.527
Lead to lead strong connection (intraprocedural diagnosis); n (%)	15	22.39%	22	11.70%	153	6.44%	0.033	<0.001	0.006
Procedure-related potential risk factors of major complications									
Extraction of ICD leads; n (%)	7	10.45%	38	20.21%	687	28.92%	0.072	<0.001	0.011
Extraction of abandoned lead; n (%)	15	22.39%	30	15.96%	214	9.01%	0.010	<0.001	<0.001
Extraction of UP lead; n (%)	24	35.82%	32	17.02%	229	9.64%	<0.001	<0.001	<0.001
Passive fixation	53	79.10	135	71.81	1316	55.38	0.316	<0.001	<0.001
Dwell time of oldest lead extracted in the patient [months]	178.8	89.48	124.8	71.20	99.44	73.64	<0.001	<0.001	<0.001
Cumulative dwell time of extracted leads; [y]	26.05	16.95	18.19	13.54	13.61	12.53	<0.001	<0.001	<0.001
Mean duration of extracted lead (in the whole group); [months]	163.6	89.61	121.5	69.10	97.14	71.61	<0.001	<0.001	<0.001
Risk of major complications calculated by SEFETY TLE calculator (%)	4.03	4.33	2.71	3.81	1.71	3.02	0.012	<0.001	<0.001
Utility of additional tools									
Evolution (old and new) or TightRail; n (%)	5	7.46%	7	3.72%	30	1.26%	0.356	0.002	0.016
Lasso catheter/snare; n (%)	15	22.39%	9	4.79%	76	3.20%	<0.001	<0.001	0.333
TLE efficacy and complications									
Major complications (any); n (%)	20	29.95%	5	2.66%	24	1.01%	<0.001	<0.001	0.089
Hemopericardium; n (%)	4	5.97	4	2.13%	21	0.86%	0.254	<0.001	0.199
Death procedure related (intra-, post-procedural); n (%)	0	0.00%	0	0.00%	0	0.00%			
Death indication-related (intra, post-procedural); n (%)	0	0.00%	0	0.00%	0	0.00%			
Partial radiological success (remained tip or <4 cm lead fragment); n (%)	11	16.42%	5	2.66%	94	3.96%	<0.001	<0.001	0.489
Full clinical success; n (%)	49	73.13%	185	98.40%	2342	98.57%	<0.001	<0.001	0.892
Full procedural success; n (%)	41	61.19%	182	96.81%	2287	96.25%	<0.001	<0.001	0.852
Death during whole FU; n (%)	10	14.93%	62	32.98%	770	30.41%	0.008	0.004	0.936

Abbreviations: ICD-implantable cardioverter defibrillator, UP-unipolar lead, TLE-transvenous lead extraction, TR-tricuspid regurgitation.

**Table 4:** Echocardiographic findings/abnormalities recorded in patients with and without TLE-related TVD.

Echocardiographic Findings/Abnormalities Recorded in Patients Undergoing TLE	TR Increased for 2 or 3 Degrees		TR Increased for 1 Degree		TR Unchanged or Even Decreased				
Number of patients number of the group	67	A	188	B	2376	C	*p*	*p*	*p*
Presented values	Count/average	%/Sd	Count/average	%/Sd	Count/average	%/Sd	A vs. B	A vs. C	B vs. C
ECHO before and after TLE									
LVEF (average)	55.93	11.40	51.88	13.54	49.13	15.51	0.030	<0.001	0.018
TR significant (3 and 4 degree)	0/67	0.00%	22/188	11.70%	511/2376	21.51%	0.003	<0.001	0.001
Any shadows on the leads before TLE									
Any shadows on leads before TLE	42/67	62.69%	114/188	60.64%	1172/2376	49.33%	0.768	0.031	0.003
Connecting tissue surrounding the lead	10/67	14.93%	23/188	12.23%	237/2376	9.98%	0.573	0.185	0.323
Thickening of the lead	26/67	38.81%	46/188	24.47%	431/2376	18.14%	0.025	<0.001	0.032
Strong connective tissue scar connection of the lead with heart structures (any)	31/64	48.44%	46/188	24.47%	261/2301	11.34%	<0.001	<0.001	<0.001
Strong connective tissue scar connection of the lead with tricuspid apparatus	26/57	45.61%	26/188	13.83%	88/2376	3.70%	<0.001	<0.001	<0.001
Strong connective tissue scar connection of the lead with RA wall	5/67	7.46%	10/188	5.32%	95/2376	4.00%	0.522	0.158	0.379
Strong connective tissue scar connection of the lead with SVC	12/67	17.91%	10/188	5.32%	91/2376	3.83%	0.002	<0.001	0.312
Strong connective tissue scar connection of the lead with RV wall	27/67	40.30%	24/188	12.77%	120/2376	5.05%	<0.001	<0.001	<0.001
Strong connection of the lead with another lead with connecting tissue scar	14/67	20.90%	26/188	13.83%	214/2376	9.01%	0.172	<0.001	0.029
Phenomena observed during monitoring of TLE procedure with TEE (only in patients monitored by TEE during TLE)									
Drawing of RA/RAA during mechanical lead extraction	22/48	45.83%	50/106	47.17%	431/2376	18.14%	0.877	<0.001	<0.001
Drawing of tricuspid leflet during mechanical lead extraction	33/48	68.75%	28/106	26.42%	64/2376	2.69%	<0.001	<0.001	<0.001
Drawing of RV wall during mechanical lead extraction	32/48	66.67%	48/106	45.28%	271/2376	11.41%	0.014	<0.001	<0.001
Abnormal lead loops visible in preoperative TTE/TEE									
Lead loops in the heart (any)/ECHO	19/66	28.79%	54/188	28.72%	408/2376	17.17%	0.992	0.014	<0.001
Loop in the RA	12/67	17.91%	39/187	20.86%	300/2376	12.63%	0.606	0.201	0.001
Loop in the TV	5/67	7.46%	16/186	8.60%	98/2376	4.13%	0.772	0.180	0.004
Loop in the RV	8/67	11.94%	14/187	7.49%	118/2376	4.97%	0.266	0.012	0.133

Abbreviations: LVEF—left ventricular ejection fraction, RA—right atrium, RAA—right atrial appendage, RV—right ventricle, SVC—superior vena cava, TLE—transvenous lead extraction, TR—tricuspid regurgitation, TTE—transthoracic echocardiography, TEE—transesophageal echocardiography.

**Table 5:** Potential risk factors of mild and severe TVD during TLE—results of multivariable linear regression step-wise analysis.

	Univariable Regression			Multivariable Regression		
	OR	95% CI	*p*	OR	95% CI	*p*
Mild TLE-related tricuspid valve damage risk factors						
Presence of abandoned lead before TLE [yes/no]	1.762	1.176–2.642	0.006	1.624	1.002–2.632	0.048
TR before TLE [by one degree]	0.610	0.501–0.743	0.001	0.546	0.443–0.674	<0.001
Strong CTS connection of the lead with tricuspid apparatus [yes/no]	4.443	2.779–7.106	<0.001	3.452	1.964–6.640	<0.001
Lead loops in the heart (any)/ECHO [yes/no]	2.001	1.430–2.799	<0.001	1.726	1.203–2.476	0.003
Severe TLE-related tricuspid valve damage risk factors						
Device type (AAI, VVI, DDD, CRT-P) [yes/no]	5.289	2.112–13.24	0.001	5.264	1.182–23.45	0.029
Dwell time of oldest one extracted lead [by one year]	1.124	1.091–1.157	<0.001	1.076	1.006–1.151	0.032
Extraction of UP lead [yes/no]	2.581	1.788–3.756	<0.001	1.776	0.971–3.245	0.062
TR before TLE [by one degree]	0.306	0.193–0.485	0.001	0.155	0.078–0.305	<0.001
Strong CTS connection of the lead with tricuspid apparatus [yes/no]	15.43	8.830–26.96	<0.001	5.720	2.378–13.75	<0.001
Strong CTS connection of the lead with RV wall [yes/no]	13.79	8.064–23.58	<0.001	8.312	3.484–19.83	<0.001

AAI—one-chamber pacemaker with atrial lead, CRT-P—cardiac resynchronization therapy pacemaker, CTS—connective scar tissue, DDD—dual chamber pacemaker, RV—right ventricle, TLE—transvenous lead extraction, TR—tricuspid valve regurgitation, UP—unipolar leads, VVI—one-chamber pacemaker with right ventricular lead.

## Data Availability

Readers can access data supporting the conclusions of the study upon reasonable request to the authors.
